# Inhibition of Pathological Increased Matrix Metalloproteinase (MMP) Activity for Improvement of Bone Regeneration in Diabetes

**DOI:** 10.3390/life12020134

**Published:** 2022-01-18

**Authors:** Johannes Maximilian Wagner, Felix Reinkemeier, Christoph Wallner, Mehran Dadras, Stephanie Dittfeld, Marius Drysch, Alexander Sogorski, Maxi von Glinski, Marcus Lehnhardt, Björn Behr, Mustafa Becerikli

**Affiliations:** Department of Plastic and Reconstructive Surgery, BG University Hospital Bergmannsheil, Ruhr-University Bochum, 44789 Bochum, Germany; max.jay.wagner@googlemail.com (J.M.W.); Felix.Reinkemeier@ruhr-uni-bochum.de (F.R.); c.wallner88@gmail.com (C.W.); mdadras@outlook.com (M.D.); dittfeld.stephanie@gmail.com (S.D.); marius.drysch1@gmx.net (M.D.); alexander.sogorski@gmx.de (A.S.); maxivonglinski@gmail.com (M.v.G.); marcus.lehnhardt@bergmannsheil.de (M.L.); bjorn.behr@rub.de (B.B.)

**Keywords:** bone regeneration, diabetes, gene expression, MMP, Marimastat

## Abstract

Patients with diabetes suffer from poor fracture healing. Molecular reasons are not fully understood and our previous gene expression microarray analyses of regenerating bones from mice with type 2 diabetes (db^−^/db^−^) revealed accelerated activation of pathways concerning matrix metalloproteases (MMPs). Thus, we picked out the pathological MMP acceleration as a target for profound gene expression analyses and additional therapeutic intervention in the present study. In the first part, gene expression of ECM degrading proteinases and inhibitors was investigated three and seven days postoperatively. *Mmp3*, *Mmp9*, *Mmp13* and gene expression of MMP inhibitor *Timp2* was significantly higher in regenerating bone fractures of db^−^/db^−^ compared to wild type animals. *Timp1* and metalloproteinase *AdamTS4* showed no differences. In the second part, we locally applied a single dose (1 µL of 5 µM solution) of the broad-spectrum molecular MMP inhibitor Marimastat on tibial defects in db^−^/db^−^. We performed immunohistochemical and histological stainings seven days post operation. Impaired bone healing, collagen content, angiogenesis, and osteoclast invasion in db^−^/db^−^ were restored significantly by application of Marimastat compared to PBS controls (*n* = 7/group). Hence, local intervention of bone defects by the molecular MMP inhibitor Marimastat might be an alternative therapeutic intervention for bone healing in diabetes.

## 1. Introduction

Patients with diabetes (PwD) without metabolically stable blood glucose (hyperglycemia) complain of disturbed bone homeostasis, increased risk of fracture occurrence, and poor healing after fractures [1,2,3,4,5,6]. Nearly 90% of PwD have type 2 diabetes [7]; it exhibits hyperglycemia due to insulin resistance [8]. Considering that diabetes concerns 13% of US adults and that an additional nearly 35% of adults are estimated to have increased blood glucose that may develop to type 2 diabetes without intervention, an ever increasing health and economic burden is looming [9]. Therefore, investigation of further options for therapy for deficient bone healing in PwD is of particular interest and clinical relevance. Thus, there is a focus on dysfunction of matrix components or associated cells.

In former studies, we exhibited a reduction in regeneration of tibial bone, osteogenic differentiation, and osteoclastic activity in mouse models of type 2 diabetes (db^−^/db^−^) [10,11]. Subsequently, we examined the profile of expressed genes in db^−^/db^−^ bone and bone of wild type mice (wt) [12]. For this purpose, tibial defects were created, and expression profile was examined in regenerating bone and uninjured bone by comparative microarray analyses. These data enable the identification of pathologically deregulated pathways and potential target factors for therapeutic intervention to restore disturbed bone fracture healing in diabetes.

Analyses of microarray data revealed activation of canonical pathways concerning inhibition of matrix metalloproteases (MMPs) in regenerating wt bones compared to regenerating bones of db^−^/db^−^ [12]. Conversely, microarray data exhibited an increased expression of MMPs in regenerating bones of db^−^/db^−^ compared to regenerating wt bone. Thus, in this present study, we selected the pathological MMP acceleration in bones of db^−^/db^−^ as a potential target for therapeutic intervention.

Bone homeostasis is a permanent process that is strictly balanced by bone formation and resorption [13]. The family of MMPs consists of highly homologous enzymes of zinc-dependent proteinases that degrade extracellular matrix (ECM) components like collagen, fibronectin, and elastin [14,15]. Relation of MMPs with bone homeostasis has become evident with the use of MMP-deficient animal models, which show a series of bone abnormalities like delayed ossification and fracture repair, anomalous bone development, and irregularly shaped bones or diminished resistance to fracture [14]. In contrast to MMP deficiency, MMP activity and over expression contribute to several bone pathologies including osteoporosis, osteonecrosis, arthritis, or bone cancer metastasis [14]. In addition to MMPs, there are also other metalloproteinases that degrade ECM components. ADAMTS4 (aggrecanase-1) belongs to the ADAMTS (a disintegrin and metalloproteinase with thrombospondin motifs) family, which can degrade aggrecan, an ECM component of cartilage [16]. Tissue inhibitors of metalloproteinase (TIMP) 1 and 2 are the most analyzed natural inhibitors of MMPs and show the highest expression during the development of bone [17]. Nevertheless, TIMP1 and TIMP2 are also able to activate bone resorption of osteoclasts and, hence, bone loss, independent of their MMP inhibiting function [18].

Thus, in the first experimental part, we investigated the gene expression of the ECM degrading proteinases *Mmp3, Mmp9, Mmp13, AdamTS*, and MMP inhibitors *Timp1* and *Timp2* in bone of wt and db^−^/db^−^ animals. Subsequently, in the second experimental part, we applied a broad-spectrum molecular MMP inhibitor (Marimastat) locally into tibial defects of db^−^/db^−^ mice and, thus, analyzed the therapeutic impact on bone physiology and regeneration. Marimastat was initially developed as an antineoplastic drug that binds covalently to the zinc atom at the MMP-active site with an IC_50_ about 2–200 nM for MMP-1, -2, -3, -7, -8, -9, -13, -14 [15,19,20,21].

## 2. Materials and Methods

### 2.1. Surgery of Animals

The Institutional Animal Care and Use Committee, LANUV NRW, confirmed the experiments (#AZ: 84-02.04.2016.A045). Mice were purchased from Jackson Laboratory. Wild type control mice (wt, C57BL/6J) and mice with diabetes (db^−^/db^−^, homozygous Lepr^db^, catalog no. #000697) were kept with standard laboratory chow and water ad libitum (12 to 16 weeks of age). Tibial defects were performed as previously described [10,11,12,22]. Briefly, mice were anesthetized with isoflurane (Abbott GmbH, Wiesbaden, Germany), and on the tibial surface, a unicortical hole (Ø 1 mm) was placed by a micromotor (Ultimate NSK 450, NSK Europe, Maidenhead, England).

**Figure 1:**
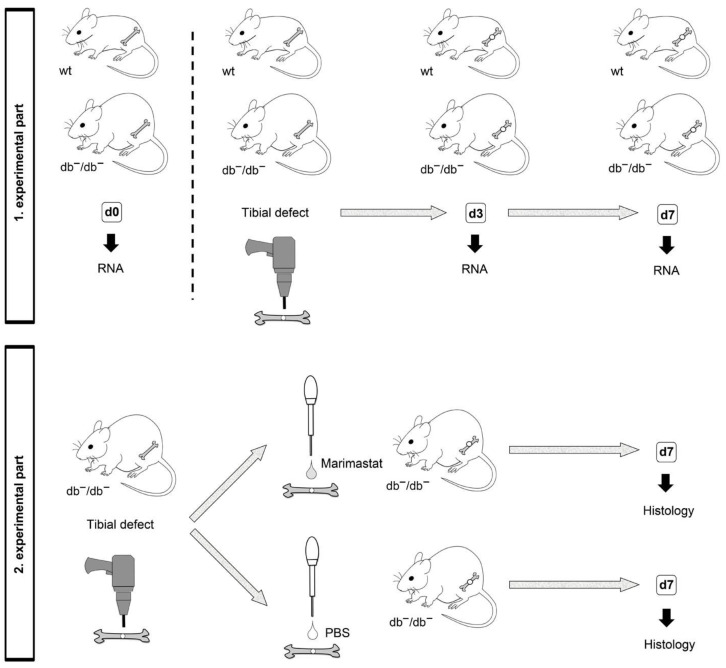
Illustration of experimental groups. wt: Wild type mouse; db^−^/db^−^: Mouse with diabetes; d0: Uninjured bone; d3: Regenerating bone three days post operation; d7: Regenerating bone seven days post operation.

In the first section of this project, in order to acquire bone tissue of wt and db^−^/db^−^ mice for gene expression assays, treatment was not performed, and the wound was closed after creating the defect. Animals were euthanized according to national and international laws and guidelines by cervical dislocation three and seven days post operation (d3 and d7), which depicts early and late time points during bone regeneration [23,24]. Mice in the uninjured bone group (d0) obtained no operation. Each experimental group consisted of at least five mice (Figure 1, top).

In the second section, treatment was performed, and only db^−^/db^−^ mice were used. Marimastat (Tocris Bioscience, Bristol, UK) stock solution (10 mM) was dissolved in PBS to a final concentration of 5 µM. The defect was closed with a collagen sponge applied with 1 µL of 5 µM Marimastat. The control group without treatment received a collagen sponge applied with 1 µL PBS. After seven days post operation (d7), euthanasia was carried out by cervical dislocation. At least seven mice were used for each experimental group (Figure 1, down).

### 2.2. RNA Isolation and Quantitative Real-Time PCR (qRT-PCR)

Tibiae were harvested quit of any soft tissue at a given time. RNA was isolated through the mirVana™ RNA Purification Kit (Life technologies, Carlsbed, CA, USA) following kit instructions and as previously described [12]. Bone fragments were taken with sizes of 2 to 3 mm from tibiae without defect or fragments only harboring the hole (5 mice/group of 1st experimental part). Additionally, 200 ng RNA/reaction was used for reverse transcription by High Capacity cDNA RT-Kit (Thermo Fisher Scientific, Waltham, MA, USA), utilizing RNase inhibitors following kit instructions. qRT-PCR was performed with 2 µL cDNA for each reaction by TaqMan^®^ assays (Supplementary Table S1 lists gene and assay IDs) with a StepOnePlus PCR System (Applied Biosystems, Waltham, MA, USA), as previously described [12], and data analysis was performed according to the manufacturer’s ΔΔCt method with *Hprt1* and *18S* as reference genes. Gene expression was illustrated in fold changes compared to d0 wt bone without defect.

### 2.3. Preparation of Tissues and Characterization of Bone Formation after Marimastat Application

At d7 post operation, tibiae of seven animals/group were collected and fixed overnight by 4% paraformaldehyde at 4 °C. Samples were decalcified with 19% EDTA (Applichem, Darmstadt, Germany) for two weeks, and samples embedded in paraffin were cut into serial sagittal sections (thickness 6–9 μm). Characterization of bone formation with aniline blue (Carl Roth, Karlsruhe, Germany) staining was performed with every sixth section, as described previously [12,22]. Briefly, pictures of entire defects were taken, and aniline blue pixels were quantified by Adobe Photoshop (Adobe, San Jose, CA, USA) semi-automatically via the magic wand tool (tolerance 60%; noncontiguous). Data are illustrated in arbitrary units.

### 2.4. Histological Analyses

Immunofluorescent and histochemical stainings were performed, as previously described [22,25]. Briefly, primary antibody against osteocalcin (rabbit, polyclonal, SantaCruz Biotechnology, sc-30045, 1:50) was applied. Secondary antibodies conjugated to HRP and NovaRED (HRP) Peroxidase Substrate Kit (Vector Laboratories, Burlingame, CA, USA) were utilized. For immunofluorescent stainings, primary antibodies against CD31 (rat, monoclonal, BD Biosciences, 553370, 1:400), MMP3 (rabbit, monoclonal, abcam, ab52915, 1:100), and collagen type I alpha 1 (Col1A1, mouse, monoclonal, SantaCruz Biotechnology, sc-293182, 1:100) were utilized. Alexa Fluor594 conjugated secondary antibody (Thermo Fisher Scientific) was used. Isotype control was done with rabbit IgG isotype control antibody (Thermo Fisher Scientific). All sections were counterstained with DAPI. Three images/samples within the defect site were taken and histomorphometry was carried out, as described previously, by semiautomatic quantification of pixels using Adobe Photoshop [12]. Data are illustrated in arbitrary units.

### 2.5. Tartrate-Resistant Acid Phosphatase (TRAP) Staining

Tartrate-resistant acid phosphatase (TRAP), mainly expressed by osteoclasts, was stained with the leucocyte acid phosphatase kit (#387A-1KT; Sigma-Aldrich, St. Louis, MO, USA) following kit instructions, as described previously [26]. Analysis was performed using Photoshop as described above, by automatically quantifying the number of all TRAP positive pixels. Data are illustrated in arbitrary units.

### 2.6. Statistical Analysis

Results are exhibited as mean ± standard deviation (SD). In the first experimental section, d0 data of wt animals were defined as 1. In the second experimental section, data of db^−^/db^−^ control mice not treated with Marimastat were defined as 100%. Statistical analyses were performed by unpaired 2-tailed Student**’**s *t*-test, using GraphPad PRISM (Graphpad Software, Inc., San Diego, CA, USA). *p*-values < 0.05 were considered statistically significant and indicated in the figures as follows: *: *p* < 0.05; **: *p* < 0.01.

## 3. Results

### 3.1. Increased Expression of MMP-Related Genes in Regenerating Bones of db^−^/db^−^ Mice

**Figure 2:**
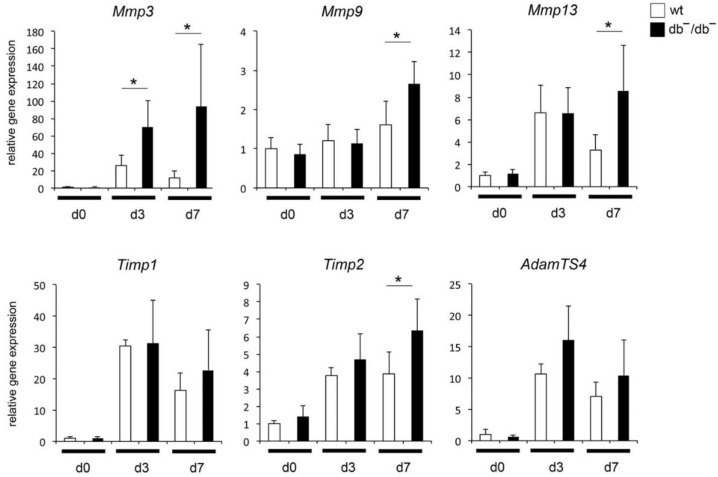
Quantitative RT-PCR analysis of *Mmp, Timp* and *AdamTS* genes in bones of wt mice and mice with diabetes (db^−^/db^−^) without injury (d0) and healing bones three and seven days post operation (d3 and d7). *Mmp: Matrix metalloproteinase; Timp: Tissue inhibitor of metalloproteinases; AdamTS: A disintegrin and metalloproteinase with thrombospondin motifs.* Results are depicted as ±SD. *p*-value: * < 0.05.

To understand the mechanisms of diminished regeneration of bones in diabetes, activation of distinct pathways in healing bones of wt and db^−^/db^−^ was examined by microarray analyses. Data revealed noticeable activation of canonical pathways concerning inhibition of MMPs in regenerating wt bones [12]. Several genes related to MMPs were selected in order to analyze gene expression by qRT-PCR in more detail. During bone regeneration, expression of *Mmp*3 and Mmp*13* was significantly elevated in general, whereas up-regulation in bones of db^−^/db^−^ was significantly higher compared to wt bones (Figure 2). Expression of *Mmp9* was not up-regulated in regenerating wt bone, whereas up-regulation in bones of db^−^/db^−^ was observed seven days (d7) postoperatively. Additionally, expression patterns of MMP inhibitors *Timp1*, *Timp2* and metalloproteinase *AdamTS4* were investigated. A significant up-regulation of gene expression of all examined genes was observed in regenerating bones, whereas up-regulation of Timp2 was significantly higher in bone of db^−^/db^−^ as compared to bone of wt animals seven days (d7) postoperatively (Figure 2).

### 3.2. Enhanced New Bone Formation by Reducing MMP Activity in Bones of db^−^/db^−^ Mice

**Figure 3:**
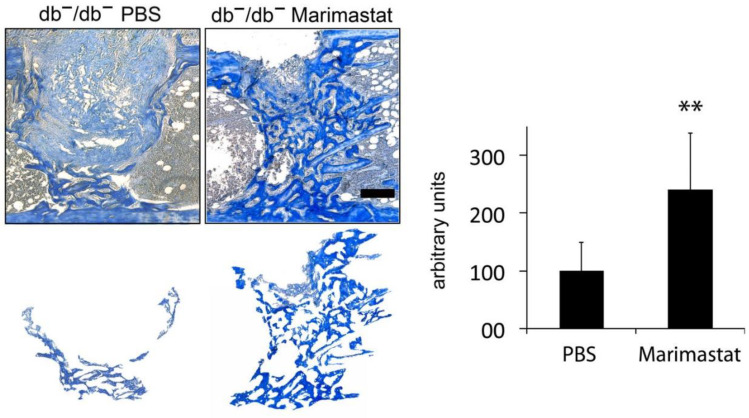
Staining of new bone formation by aniline blue. Diminished formation of new bone in tibial defects of mice with diabetes (db^−^/db^−^) was recovered by local Marimastat therapy seven days post operation. Aniline blue positive pixels were extracted and quantified. Improvement of bone formation was observed (2.4× times). Values are depicted as ±SD. *p*-value: ** < 0.01. Scale bar: 200 μm.

Osteogenesis is significantly impaired in bones of db^−^/db^−^. Gene expression profiles quantified and evaluated by microarray analyses and qRT-PCR measurement exhibited enhanced activation of pathways concerning MMP activity in healing bones of animals with diabetes compared to wt controls. Consequently, we picked out pathologic MMP activity as a therapeutic target in the present study. To investigate the regenerative potential of inhibiting MMP activity by Marimastat, formation of osteoid in tibial defects in treated and untreated animals with diabetes was compared seven days post operation. Diminished regeneration of bone in diabetes was reconstituted, and formation of new bone was enhanced 2.4× times in db^−^/db^−^ animals, by inhibiting MMP activity via Marimastat application (Figure 3).

### 3.3. Enhanced Collagen Content by Inhibition of MMP Activity in Bones of db^−^/db^−^ Mice

**Figure 4:**
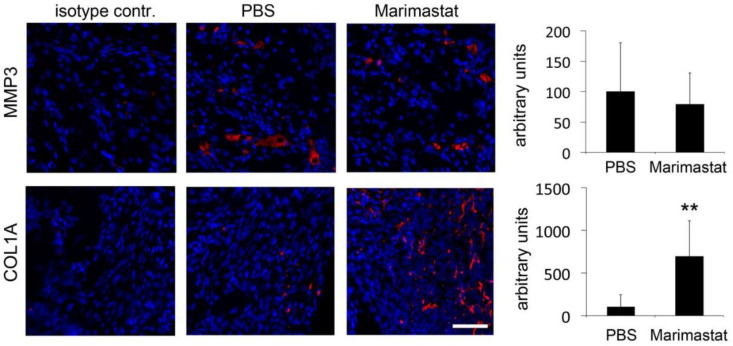
Immunohistochemistry for MMP3 and COL1A1. MMP3 content was not affected, whereas an increase in COL1A1 level was detected in healing bones of db^−^/db^−^ mice after inhibition of MMP activity seven days postoperatively. Values are depicted as ±SD. *p*-value: ** < 0.01. Scale bar: 50 μm.

Regenerating bones of db^−^/db^−^ mice exhibited decreased collagen content [27,28]. The most abundant collagen is type 1; it is present in all connective tissues and is the main structural protein of bones [29]. Representatively, we analyzed the content of MMP3 and COL1A1, one of the components of type I collagen, after inhibiting ECM degradation activity of MMPs by Marimastat. MMP3 level was not affected by Marimastat, whereas COL1A1 content was significantly increased seven days postoperatively (Figure 4).

### 3.4. No Observable Effect of Marimastat on Osteocalcin Level Seven Days Postoperatively

**Figure 5:**
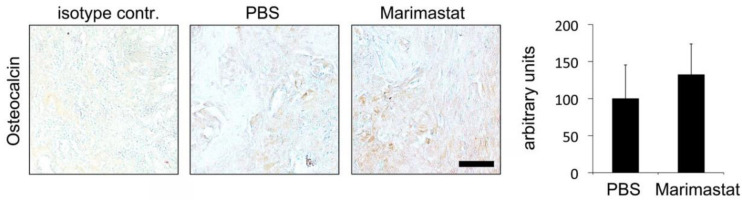
Immunohistochemistry for osteocalcin. An increase in osteocalcin level was detected in healing bones of db^−^/db^−^ mice after inhibition of MMP activity seven days postoperatively, albeit it was not significant. Values are depicted as ±SD. Scale bar: 50 μm.

We previously demonstrated that osteoblast activity diminished in db^−^/db^−^ mice [10,12,22]. In order to analyze osteoblast activity in db^−^/db^−^ mice after Marimastat application, immunohistochemical staining of osteocalcin (protein secreted by osteoblasts that is necessary for bone mineralization) was performed. Osteocalcin levels increased, albeit not significantly (Figure 5).

### 3.5. Enhanced Angiogenesis by Inhibition of MMP Activity in Bones of db^−^/db^−^ Mice

**Figure 6:**
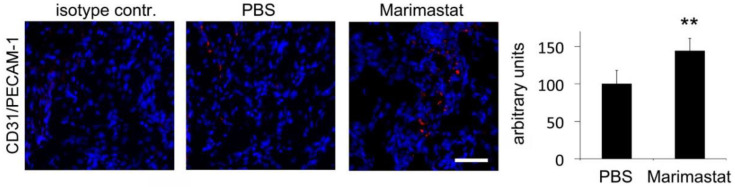
Immunohistochemical staining of CD31 (platelet endothelial cell adhesion molecule 1, PECAM-1. Diminished angiogenesis in regenerating bone of mice with diabetes was slightly reconstituted by inhibition of MMP activity. Values are depicted as ±SD. *p*-value: ** < 0.01. Scale bar: 50 μm.

Previous experiments exhibited decreased angiogenesis in bony defects of mice with diabetes [10,12,22]. In order to analyze blood vessel formation after local Marimastat treatment, immunofluorescent staining of CD31/PECAM-1 was performed in regenerating bone of db^−^/db^−^ seven days postoperatively. A significant increase in CD31/PECAM-1 level was detected after inhibition of MMP activity by Marimastat (Figure 6).

### 3.6. Increased Osteoclast Invasion by Inhibition of MMP Activity in Bones of db^−^/db^−^ Mice

**Figure 7:**
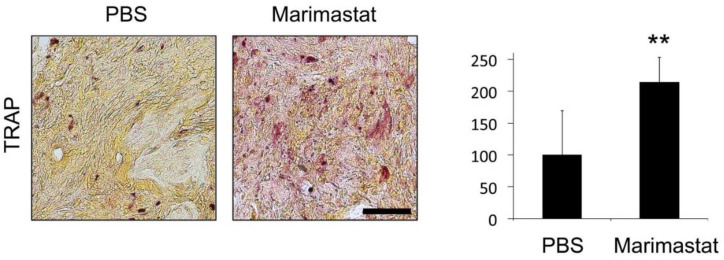
TRAP staining for osteoclast invasion. Impaired osteoclast invasion in regenerating bones of mice with diabetes was recovered by inhibition of MMP activity. Values are depicted as ±SD. *p*-value: ** < 0.01. Scale bar: 50 μm.

Further experiments showed impaired osteoclast invasion into bone defects of mice with diabetes [10,12,22]. TRAP is highly expressed in osteoclasts and serves as an osteoclastic marker. TRAP-staining revealed increased osteoclast invasion in damaged bone areas of Marimastat-treated db^−^/db^−^ mice seven days postoperatively (Figure 7). Thus, diminished osteoclast levels rebounded to physiological levels in regenerating bones of mice with diabetes after inhibition of MMP activity by Marimastat.

## 4. Discussion

Bone tissue undergoes constant remodeling, in which old tissue is replaced and defects are restored. Impaired fracture healing and bone regeneration is determined during diabetes. To explore the molecular mechanisms leading to this finding, we examined the gene expression profiles of uninjured and regenerating bone tissues from wt animals and animals with diabetes, by microarray analyses in a recent study [12]. In healing non-pathologic wt bone, canonical pathways associated with inhibition of MMPs were one of the top significantly activated pathways. Conversely, *Mmp3* gene expression was one of the top elevated gene transcripts in healing bone of animals with diabetes in these microarray analyses.

The balance between MMPs and their inhibitors, TIMPs, is essential for matrix remodeling processes during bone regeneration and is crucial for assuring bone quality. Thus, MMP degradation results in a decrease in bone mass, while TIMP overexpression leads to an increase in bone mass [30]. Nevertheless, over-expression of MMPs or higher level of MMP activation is frequently reported in cardiovascular diseases like atherosclerosis or joint disorders such as osteoarthritis (OA) or rheumatoid arthritis (RA). OA and RA are characterized by excessive reduction and loss of cartilage ECM, which ultimately result in the persisting remodeling of joints [31,32].

MMP3 and MMP13 are the most strongly associated MMPs in destruction of cartilage in human OA [32]. Furthermore, MMP3 is a useful marker for predicting cartilage and bone damage in RA [33]. MMP9 is primarily expressed by inflammatory cells during complaints such as RA and cancer [34].

In the present study, validation of our previously published microarray results by further qRT-PCR analysis revealed an up-regulation of *Mmp3* and *Mmp13* gene expressions in regenerating bone fractures three and seven days (d3, d7) postoperatively, whereas up-regulation in bone of db^−^/db^−^ compared to bone of wt mice was significantly higher. Furthermore, expression of *Mmp9* was up-regulated in regenerating bone of db^−^/db^−^ seven days (d7) postoperatively.

Moreover, due to their broad actions, MMPs are also involved in proliferation, differentiation, cell attachment, or apoptosis of chondrocytes in growth plates, as well as in the regulation of biological activity of growth factors and their receptors, cytokines, and a variety of enzymes [35]. An activation of TGFb by MMPs is also described [36]. Interestingly, excess TGFb pathway activity is also associated with impaired bone regeneration in diabetes **[12]**.

Similar to MMPs, ADAMTSs have been associated with degradation of tissue and inflammation [37]. Nevertheless, high expression levels of *AdamTS4* during chondrogenic differentiation of mesenchymal stem cells (MSCs) were observed [38]. The balanced activity of natural inhibitors of MMPs, TIMPs, is fundamental for normal bone homeostasis and assuring bone quality. Nevertheless, a negative regulatory role of TIMP1 in the proliferation and osteogenesis of bone marrow-derived mesenchymal stromal cells (BMSCs) was described previously [39]. In the present study, expression of *AdamTS4* and *Timp1* was up-regulated in regenerating bone fractures of wt and db^−^/db^−^ generally, whereas up-regulation in bones of mice with diabetes was statistically not significantly higher than in wt bone. Thus, the activity of these enzymes was according to physiological range. The knowledge of TIMP2 function in bones of PwD is quite sparse. Diabetes and OA often co-exist in older adults [40], and TIMP2 is involved in cartilage destruction in OA [41]. Thus, appropriate activity of ECM degrading enzymes in the right place at the right time is required during physiological bone regeneration.

Although most studies show that inhibition of MMPs by Marimastat blocks angiogenesis, there are also studies demonstrating the opposite. Stabilization of microvessels mediated by Marimastat was associated with blockade of collagen lysis and accumulation of collagen fibrils [42]. Similarly, we also observed an increased microvessel density after MMP inhibition seven days postoperatively. Consequently, in order to analyze whether this increase was also associated with inhibition of collagen lysis or accumulation of collagen fibrils, we representatively analyzed the content of MMP3 and COL1A1 after inhibiting ECM degradation activity of MMPs by Marimastat. An alteration in MMP3 level was not detected by immunofluorescent staining. Thus, Marimastat binds the MMP-active site presumably without affecting MMP level or shielding the protein from binding by antibodies during IHC. In contrast, inhibition of broad MMP activity significantly increased COL1A1 content by protecting collagens from lysis.

Additionally, in the present study, protein levels of osteocalcin, which is secreted by osteoblasts in association with bone mineralization, showed no significant differences in Marimastat treated and untreated defects seven days postoperatively. Nevertheless, a significant increase in osteocalcin level over several days postoperatively, after Marimastat treatment with subsequent decrease after seven days, would be possible.

Further studies on bone defects in animals with diabetes also revealed impaired fracture healing. Similar to our experiments, regeneration of defects of femoral bone was significantly delayed at days 7 and 10 in mice with type 1 diabetes. Additionally, bone marrow density and osteoclast invasion measured by TRAP staining were also significantly impaired [43]. Analyzes that go beyond the examination time of 10 days also showed impaired formation of new bone area 12, 16, and 22 days post operation [44]. Moreover, increased gene expression of *AdamTS4* and *Mmp13* was also observed on days 16 and 22, respectively [44]. Thus, pathological increased metalloproteinase activity that is responsible for degradation of ECM components is not a temporary obstacle that disappears after one week. Consequently, bone regeneration and physiology are impaired at least more than three weeks, and a targeted therapeutic intervention can improve bone regeneration efficiently, as demonstrated in the present study.

In further studies, we also applied recombinant fibroblast growth factor-9 (FGF-9), vascular endothelial growth factor A (VEGFA), or isogenic adipose derived stem cells (mASCs) in tibial defects of db^−^/db^−^ animals. Application enhanced osteogenesis, angiogenesis, and bone remodeling in regenerating bones of mice with diabetes [10,22].

In summary, examination of gene expression in regenerating bones of mice with diabetes during fracture healing exposed an enhanced MMP activity compared to wt bone. A local therapy with the molecular MMP-inhibitor Marimastat resulted in improved fracture healing. Because the MMP function is well conserved in vertebrates, we anticipate that MMP-mediated molecular therapies that directly target bone homeostasis in human PwD could be therapeutic.

## 5. Conclusions

Diabetes care is evolving into an ever-increasing health and economic burden. Any approach that can help to tackle this task should be taken into consideration. From a clinical point of view, disturbance of bone fracture healing belongs to one of the countless complications occurring due to hyperglycemia, whereas the molecular reasons are not fully understood so far. In this study, we demonstrated that gene expression data of regenerating bones could provide important information in determining components responsible for dysfunctions in bone fracture healing. In this context, we showed that accelerated MMP activity is one of the components and that a therapeutic intervention by inhibiting this pathologically elevated MMP activity improves bone regeneration.

## Data Availability

The data that support the findings of this study are available in the figures, tables and the Supplementary Material of this article.

## Supplementary Materials

The following are available online at https://www.mdpi.com/article/10.3390/life12020134/s1, Table S1: Genes and TaqMan^®^ gene expression assay Ids.
